# Changes in TNFα, NFκB and MnSOD protein in the vestibular nuclei after unilateral vestibular deafferentation

**DOI:** 10.1186/1742-2094-7-91

**Published:** 2010-12-09

**Authors:** Martine Liberge, Christine Manrique, Laurence Bernard-Demanze, Michel Lacour

**Affiliations:** 1 Université Aix-Marseille, UMR 6149 Université de Provence/CNRS, Neurobiologie Intégrative et Adaptative, Pôle 3C, Comportement, Cerveau, Cognition, Centre de St Charles, Case B, 3 Place Victor Hugo, 13331 Marseille Cedex 3, France

## Abstract

**Background:**

Unilateral vestibular deafferentation results in strong microglial and astroglial activation in the vestibular nuclei (VN) that could be due to an inflammatory response. This study was aimed at determining if markers of inflammation are upregulated in the VN after chemical unilateral labyrinthectomy (UL) in the rat, and if the inflammatory response, if any, induces the expression of neuroprotective factors that could promote the plasticity mechanisms involved in the vestibular compensation process. The expressions of inflammatory and neuroprotective factors after chemical or mechanical UL were also compared to verify that the inflammatory response was not due to the toxicity of sodium arsanilate.

**Methods:**

Immunohistological investigations combined the labeling of tumor necrosis factor α (TNFα), as a marker of the VN inflammatory response, and of nuclear transcription factor κB (NFκB) and manganese superoxide dismutase (MnSOD), as markers of neuroprotection that could be expressed in the VN because of inflammation. Immunoreactivity (Ir) of the VN cells was quantified in the VN complex of rats. Behavioral investigations were performed to assess the functional recovery process, including both static (support surface) and dynamic (air-righting and landing reflexes) postural tests.

**Results:**

Chemical UL (arsanilate transtympanic injection) induced a significant increase in the number of TNFα-Ir cells in the medial and inferior VN on both sides. These changes were detectable as early as 4 h after vestibular lesion, persisted at 1 day, and regained nearly normal values at 3 days. The early increase in TNFα expression was followed by a slightly delayed upregulation of NFκB 8 h after chemical UL, peaking at 1 day, and regaining control values 3 days later. By contrast, upregulation of MnSOD was more strongly delayed (1 day), with a peak at 3 days, and a return to control values at 15 days. Similar changes of TNFα, NFκB, and MnSOD expression were found in rats submitted to mechanical UL. Behavioral observations showed strong posturo-locomotor deficits early after chemical UL (1 day) and a complete functional recovery 6 weeks later.

**Conclusions:**

Our results suggest that the upregulation of inflammatory and neuroprotective factors after vestibular deafferentation in the VN may constitute a favorable neuronal environment for the vestibular compensation process.

## Background

Vestibular compensation, i.e. functional recovery after a unilateral lesion of the peripheral vestibular system, is a good model to study the cellular and molecular mechanisms involved in the neuroplasticity of the adult central nervous system. Unilateral loss of vestibular inputs to the brainstem vestibular nuclei (VN) is caused by lesioning the peripheral sensory receptors (chemical or mechanical unilateral labyrinthectomy, UL) or unilaterally transecting the vestibular nerve (UVN). These procedures produce a complex vestibular syndrome made of static and dynamic signs. The static signs include postural deficits (increased support surface, head tilt) and ocular motor deficits (spontaneous nystagmus) that are compensated within a few days or weeks, depending on the species and on the type of vestibular deafferentation. Recent findings in the cat indicated that vestibular deficits resulting from a functional deafferentation are compensated faster (2 weeks after mechanical UL) than those induced by an anatomical deafferentation (about 2 months after UVN) [1]. On the contrary, the dynamic signs (vestibulo-ocular reflex asymmetry, postural instability and equilibrium) are compensated much less completely or over a longer time [2,3].

Several hypotheses have been proposed to explain the vestibular compensation process [2,4]. At least part of the behavioral recovery process has been attributed to plasticity of neuronal and non-neuronal elements in the deafferented VN. In line, recent findings have demonstrated reactive neurogenesis in the adult cat [5,6] and a strong microglial and astroglial reaction [5,7-9] after unilateral deafferentation in the deafferented VN. Campos-Torres et al. [9] suggested that the glial reaction may be due to modifications of the environment of VN neurons and most probably to the central inflammatory tissue reaction consecutive to UL. Despite this inflammatory reaction, the central vestibular neurons did not show any sign of apoptosis after the permanent loss of their peripheral inputs [8]. Glial reaction in the vestibular nuclei may be a beneficial mechanism. It could both protect the injured tissue from long-lasting inflammation and promote the survival of deafferented vestibular neurons [9] or of newly generated neurons [6]. Finally, the inflammatory reaction could help in the vestibular compensation process.

Upon insult or infection, the brain inflammatory response generally involves the production of proinflammatory cytokines such as tumor necrosis factor α (TNFα), interleukins (IL1β, IL-6...), interferon gamma (IFN-γ), and various reactive oxygen and nitrogen species [10]. To date, the cellular inflammatory mediators produced by central vestibular glial cells and/or neurons after peripheral vestibular loss have not been characterized.

Among the inflammatory mediators, TNFα is a prominent effector of the immune response in the brain. It is constitutively expressed by astrocytes, microglia, and neurons in the normal brain [11] and upregulated following various manifestations of brain injury, including neurodegenerative disorders (Alzheimer's and Parkinson's diseases, multiple sclerosis), brain trauma, and ischemic injury (for review, see [12]). It has been suggested TNFα may thus have a pathogenic role in these disorders. Nevertheless, TNFα has been shown to exert both neurotoxic and neuroprotective effects in experimental models. TNFα can mediate cell death [13,14] but, conversely, TNFα is thought to enhance neuroprotective processes in models of acute neurodegeneration (ischemia, excitotoxicity, axotomy) and chronic (Alzheimer's disease) neurodegeneration [15-20].

Increased levels of TNFα protect cells against damage from cytotoxic insults in part via the induction of cytoprotective genes, including Manganese Superoxide Dismutase (MnSOD) [21-23]. The antioxidant enzyme MnSOD is critical for neuronal survival in various paradigms of stress-induced brain injury [23-26]. Xu et al. [27] demonstrated that the presence of an intronic nuclear factor κB (NFκB) site in the MnSOD gene was essential for the induction of MnSOD by TNFα. NFκB is a nuclear transcription factor that has a protective role in many tissues in response to stress, inflammation, and immunity. NFκB protects neurons from toxic insults and promotes neuronal growth and synaptic plasticity [24,28-30]. Widera et al. [31] demonstrated that NFκB plays a crucial role in the proliferation of neural stem cells induced by TNFα.

The present study aimed to determine whether a chemical UL (transtympanic injection of sodium arsanilate) inflicts an inflammatory response in the vestibular nuclei. This reaction could be expressed by an increased production of proinflammatory cytokines such as TNFα and could induce a compensatory change in the expression of the neuroprotective factors NFκB and MnSOD during the course of the vestibular recovery. This was achieved using immunohistochemistry, notably by quantifying modulation of TNFα, NFκB, and MnSOD labeling in the four main ipsilateral and contralateral VN (medial, superior, lateral, and inferior). The immunohistochemical data were recorded in groups of rats killed 4 h, 8 h, 1 day, 3 days, 7 days, and 15 days after chemical UL. In addition, functional recovery after chemical UL was tested in another group of rats.

TNFα, NFκB, and MnSOD labeling was also quantified in the ipsilateral and contralateral medial (MVN) and inferior (IVN) nuclei in groups of rats submitted to mechanical UL and sacrificed 8 h, 1, and 7 days after the lesion. Effects of mechanical and chemical UL were compared to ensure that arsanilate-induced changes in the expression of inflammatory and neuroprotective factors were not due to the toxicity of arsanilate on brainstem structures.

## Methods

### Animals care and housing

The experiments were performed on male adult Long Evans rats (300-350 g) obtained from R. Janvier (Le Genest-St-Isle, France). Animals were maintained on a 12 h light/dark cycle at a constant temperature (22°C) with ad libitum access to food and water. The experiments were performed in accordance with the requirements of the French "Ministère de l'Agriculture et de la Pêche" Décret no. 87-848, October 19, 1987 and to the European Communities Council Directive of November 24, 1986 (86/609/EEC). Every attempt was made to minimize both the number and suffering of animals used in these experiments.

### Vestibular lesions

Chemical UL was produced by a unilateral transtympanic injection of the toxin, sodium arsanilate. Sodium arsanilate (Sigma, St Louis, MO) was dissolved in 0.3 M sodium carbonate at the concentration of 300 mg/ml and titrated to pH 7.5.

The rats were anesthetized with an intramuscular injection of a solution of ketamine (Ketamine 1000, Virbac, Carros, France, 62.5 mg/kg) and medetomidine (Domitor, Orion Pharma, Espoo, Finland, 0.4 mg/kg). Guided through a dissecting microscope, a 26-gauge needle of injection was advanced through the tympanic membrane, and 100 μl of sodium arsanilate was infused in the left middle-ear. After the injection needle was withdrawn, the external ear canal was packed with sterile hemostatic sponge to prevent leakage of the toxin away from the injection site. When the surgery was completed, the effects of medetomidine were reversed by a subcutaneous injection of the specific antagonist atipamezole (0.33 mg/kg) (Pfizer, Paris, France). Using this protocol, the effects of anesthesia were rapidly reversed and, a few minutes later, the UL rats were able to stand and thereafter to behave nearly normally.

The mechanical UL was performed using a retroauricular approach. The entire length of the ventral edge of the left meatus was destroyed. The tympanic membrane, malleus, incus, and the stapes were removed to expose the pterygopalatine. This artery was coagulated using an electrocoagulator and the oval window was opened. Finally, the vestibule was drilled and aspirated using a suction tube. A postoperative i.m Terramycin (Pfizer, Paris, France) injection was given to prevent infection.

### Histological preparation

Thirty rats were subjected to a chemical UL. They received unilateral transtympanic injection of sodium arsanilate on the left side and were killed at six recovery times after vestibular lesion: 4 h (n = 5), 8 h (n = 5), 1 day (n = 5), 3 days (n = 5), 7 days (n = 5), 15 days (n = 5). Sham-operated rats (n = 24, four rats per group) received a unilateral transtympanic injection of 0.9% sterile saline and were killed at the same recovery time as lesioned animals.

Six rats were submitted to mechanical UL on the left side. Based on data recorded in the chemical UL rats, they were killed at three recovery times: 8 h (n = 2), 1 day (n = 2), 7 days (n = 2).

Rats were deeply anesthetized with sodium pentobarbital (100 mg/kg IP) and perfused intracardiacally with 100 ml of 0.9% saline followed by 400 ml of 4% paraformaldehyde (Sigma, St Louis, MO) in 0.1 M phosphate buffer (PB), pH 7.4. The brains were rapidly removed and postfixed in a 4% paraformaldehyde solution for 24 h, cryoprotected by successive transfers into increasing concentrations (10%, 20%, and 30%) of sucrose solution in 0.1 M PB for 72 h at 4°C. Brains were then rapidly frozen on powdered dry ice (CO_2 _gas) and kept at -80°C until use. They were sectioned coronally at 30 μm on a cryostat, and the sections were stored into stock solution (0.1 M PB (pH 7.4), 30% (v/v) glycerol and 30% (v/v) ethylene glycol) at -20°C until immunohistochemistry experiments.

### Immunohistochemistry (IHC)

Free-floating sections were rinsed three times with 0.1 M phosphate buffer saline (PBS) for 5 min, preincubated in a PBS 0.1 M solution containing 0.25% Triton X-100 and 10% bovine serum albumin (BSA) for 1 h at room temperature. Thereafter, sections were incubated under continuous agitation 24 h at 4°C in either a goat polyclonal TNFα antibody (1:200), a rabbit polyclonal NFκB antibody (1:200), or a rabbit monoclonal MnSOD antibody (1:200), diluted in a PBS 0.1 M solution containing 0.25% Triton X-100 and 2% BSA. The antibodies for TNFα and NFκB were obtained from Santa Cruz Biotechnology (Santa Cruz, CA), anti-MnSOD from Epitomic (Burlingame, CA).

On the following day, sections were washed 3 × 5 min in PBS 0.1 M, BSA 2% and 3 × 5 min in PBS 0.1 M, BSA 5%. Then, they were incubated in 1:200 biotinylated secondary antibody diluted in PBS 0.1 M, 2% BSA for 1 h at room temperature. Sections then underwent another round of washing with PBS 0.1 M, BSA 2% (3 × 5 min) and with PBS 0.1 M, BSA 5% (3 × 5 min) and were incubated in biotinylated horseradish peroxidase avidin (Vector Laboratories, Burlingame, CA) for 1 h at room temperature. Finally, for visualization, sections were reacted using 0.2 mg/ml diaminobenzidine and 0.03% hydrogen peroxide in PBS 0.1 M. After being washed with PB, the sections were mounted on microscope slides and allowed to dry overnight. The following day, sections were dehydrated in ascending series of alcohol concentrations (70%, 80%, 90%, and 100%), cleared with xylene, and coverslipped with DPX mounting media.

Immunolabeling for TNFα, NFκB, and MnSOD was examined 4 h, 8 h, 1, 3, 7 and 15 days after chemical UL, and 8 h, 1 and 7 days after mechanical UL. The specificity of the immunolabeling was validated by omission of the primary antibody. No labeling was observed in these control conditions.

### Data quantification of TNFα, NFκB, and MnSOD immunoreactivity in the vestibular nuclei

The vestibular nuclei were identified according to Paxinos and Watson's stereotaxic atlas of the rat [32]. The four VN (medial, superior, lateral, and inferior; hereinafter MVN, SVN, LVN, and IVN respectively) were easily differentiated without counterstaining. Data quantification in the MVN, SVN, LVN, and IVN was done in serial sections collected from the rostral (-9.96 mm) to the caudal (-11.76 mm) part of the brainstem. Stained cells were counted in the MVN (27 sections per rat), SVN (24 sections), LVN (15 sections), and IVN (24 sections). The variable number of sections depended on the rostrocaudal extent of the nuclei [5]. The immunoreactivity quantification was performed on both sides.

The number of cells expressing TNFα, NFκB, or MnSOD was quantified via computer-assisted image analysis using a DMLB microscope (Leica Microsystems, Wetzlar, Germany) equipped with a DXM 1200 Nikon high-resolution digital camera (1024-1024 pixels; Nikon, Tokyo, Japan) interfaced to a PC computer employing image software for capturing and processing the images (Lucia G, Nikon, Champigny-sur-Marne, France). Quantification of the labeled cells was performed via a grey-level method by adjusting a threshold brightness value, and only cells labeled with a grey value above this threshold were taken into account. Reproducibility was assessed by comparing the same data analyzed independently by two researchers blind to the animal groups. Specific labeling was quantified in each section as the number of labeled cells and was automatically computed and expressed as the mean (± SEM) for each side, each rat, and each subgroup of rats [5]. To eliminate quantification problems due to potentially asymmetric slides, data were collected from symmetrical slides only. The absence of structural asymmetry in the slides was verified on slides stained with cresyl violet.

### Behavioral investigations

Behavioral investigations were performed in another group of rats (n = 6) to assess static and dynamic postural deficits after arsanilate lesion and the time course of the functional recovery.

Static postural deficits and recovery were evaluated by measuring the surface delimited by the four legs of the rat standing erect at rest, without walking. Support surface is a very sensitive parameter used for evaluating vestibular lesion-induced static posture deficit and recovery. To quantify the support surface, the rats were placed in a box equipped with a transparent bottom and filmed from under this box. A scale drawn on the bottom served to take measurements of the four paws location. Four repeated measurements were taken for each rat before chemical UL, and at regular intervals during the course of recovery until complete recovery (60 days). The support surface was measured using an image analysis system (Canvas 9TM, Deneba Software, Miami, FL). An average was calculated for each postlesion time. Data recorded after vestibular lesions were compared to the mean prelesion values using individual references, that is, each animal acted as its own control.

Dynamic postural deficits and recovery were evaluated in the same group of rats (n = 6) using two dynamic reflex tests, the air-righting reflex and the landing reflex. The air-righting reflex was used to test the rat's ability to right itself in the air, while the landing reflex was used to test the response of the rat to a vertical linear acceleration. The tests were considered to evaluate the level of the vestibular deficit and of the recovery process following the vestibular lesion.

During the air-righting test, the rats were held at 50 cm above a cushion, in supine position. The experimenter removed his/her hands as quickly and simultaneously as possible. In a normal rat, just after the drop, the vestibular system detects a change in linear acceleration that triggers the head turning, which in turn induces the sequence of body repositioning. After vestibular lesion, the righting response is totally abolished and it recovers with time through the vestibular compensation process. A score of "0" was assigned when the air-righting reflex was complete, "2" when the animal failed to return itself and fell on its back, and "1" when it showed a partial reaction only.

During the landing reflex, the rats were held by the base of the tail and lowered toward the ground. Animals with intact vestibular function flex their neck and extend their forelimbs as they approach the surface, whereas rats with vestibular lesion at an uncompensated stage show the right response only when their forepaws or vibrissae touch the ground. A score of "0" was assigned when the animals responded properly, and "2" if ground contact was required.

The scores for the air-righting reflex and the landing reflex were summed, providing a global score of dynamic vestibular function and recovery. A zero score indicated a normal vestibular function as observed before the vestibular lesion, or a complete recovery after UL. Scores ranging from 1 to 4 pointed to vestibular dysfunction and more or less complete vestibular compensation. Three repeated measurements were taken for statokinetic tests for each rat before chemical UL and at each postoperative time. A mean was calculated for each postlesion time and compared to the mean prelesion values. As for the support surface evaluation, each animal acted as its own control.

For some experimental animals, double-blind quantification was performed for the dynamic reflex tests, which did not show any dependence on the experimentator when he/she was trained to perform the tests.

### Statistical analysis

TNFα, NFκB, and MnSOD immunolabeling in the VN observed after a chemical UL was analyzed using repeated-measures analysis of variance (ANOVAs) with lesion (sham vs arsanilate-lesioned rats) and postoperative recovery period (4, 8 h, and 1, 3, 7, 15 days) as between-factors, and with the nuclei (MVN, SVN, LVN and IVN) as within factors. When a significant difference was revealed, Student's t-tests were used for further two by two comparisons between sham and lesioned animals at the six postoperative times. The levels of significance were set at p < 0.01 (TNFα and NFκB) and p < 0.0083 (MnSOD) according to the number of multiple comparisons (ie, Bonferroni's principle).

TNFα, NFκB, and MnSOD immunolabeling in the MVN and IVN after mechanical and chemical UL was analyzed using repeated-measures ANOVA with type of lesion (mechanical vs chemical UL) and postoperative time (8 h, 1 and 7 days) as between-factors, and with the vestibular nuclei (MVN and IVN) as within factors. Unpaired t-tests were used for comparisons between mechanical and chemical UL animals at the three postoperative times. The levels of significance were set at p < 0.016 (TNFα, NFκB, and MnSOD) according to the Bonferroni's principle.

Support surface values at each postoperative time were compared with preoperative surface values for each rat by a paired Student's t-test. The global score of the rats in the air-righting and landing reflex tests recorded at each postoperative time was compared to the preoperative performance of each rat by a paired Student's t-test. The levels of significance were set at p < 0.0038 (support surface and global score) according to the number of multiple comparaisons (Bonferroni's principle).

## Results

### 1. Immunohistochemical data

#### Chemical UL-induced change in TNFα immunoreactivity in the vestibular nuclei

Repeated-measures ANOVA of the cell counts of TNFα-positive cells demonstrated that the lesion (p < 0.0001), the structure (MVN, SVN, LVN and IVN) (p < 0.0001), and the postoperative time (4 h, 8 h, 1, 3, 7 and 15 days) (p < 0.05) constituted the main fixed effects providing the sources of variation among animals. This was also corroborated by the significant interaction between these variables (p < 0.0001). In contrast, no significant differences were found between the ipsilateral and contralateral VN for both the sham-operated and the arsanilate-lesioned rats. Consequently, the data from both sides were pooled and averaged for both groups of rats.

**Figure 1:**
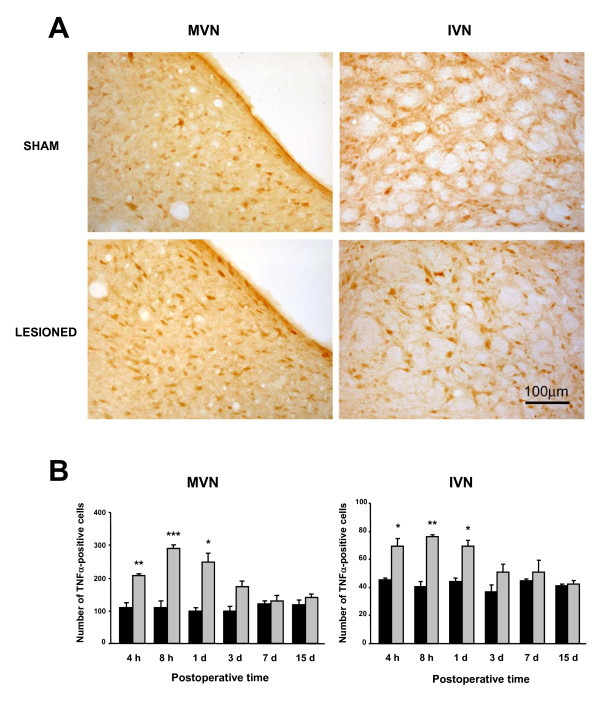
**Chemical UL-induced changes of TNFα-Ir cells in the MVN and IVN**. (A) Illustration of the typical labeling in a representative sham rat and in an experimental animal subjected to chemical UL (arsanilate transtympanic injection) observed 8 h after the lesion. Note that chemical UL induces an increased number of TNFα-Ir cells. Scare bar: 100 μm. (B) Quantitative analysis of the effects of chemical UL on TNFα-Ir cells in the MVN and IVN. Data are mean values (± SEM) of the number of TNFα-Ir recorded in the sham rats (black histograms) and in the UL rats (grey histograms) observed at 4, 8 h, 1, 3, 7, and 15 days after the lesion. The data from both sides were pooled for sham and experimental animals. According to Bonferroni's principle, P < 0.01 was considered significant. * P < 0.01, ** P < 0.001, *** P < 0.0001 versus sham values (MVN: medial vestibular nucleus; IVN: inferior vestibular nucleus).

Figure 1A illustrates the TNFα-Ir changes in the MVN and the IVN of two representative rats: one sham-operated rat and one arsanilate-lesioned rat killed 8 h after the vestibular lesion. Chemical UL resulted in an increase in TNFα expression in the both VN, as seen by the increased number of TNFα-Ir cells. This change in TNFα expression was also observed in the SVN, but not in the LVN.

Quantitative analysis of TNFα-like immunolabeling in the MVN and the IVN is shown in Figure 1B. A significantly increased number of labeled cells was first detected 4 h following the lesion, but the highest significant numbers of TNFα-positive cells were encountered in the MVN (288.6 ± 11.9, p < 0.0001) and IVN (75.9 ± 1.2, p < 0.001) at the 8 h survival period. One day after the lesion, TNFα-like immunoreactivity persisted significantly (249.5 ± 25.4 (p < 0.01) and 69.4 ± 4.5 (p < 0.01) for the MVN and IVN, respectively).

The mean number of TNFα-Ir cells regained normal values three days after the lesion (173.8 ± 16.4 and 50.9 ± 5.6, in the MVN and IVN respectively).

#### Chemical UL-induced change in NFκB immunoreactivity in the vestibular nuclei

Repeated-ANOVA revealed significant effects of the lesion (p < 0.05) and an interaction between the lesion and the postoperative time (p < 0.05). Data obtained in the sham-operated groups at the different postoperative times were pooled and considered as control values.

**Figure 2:**
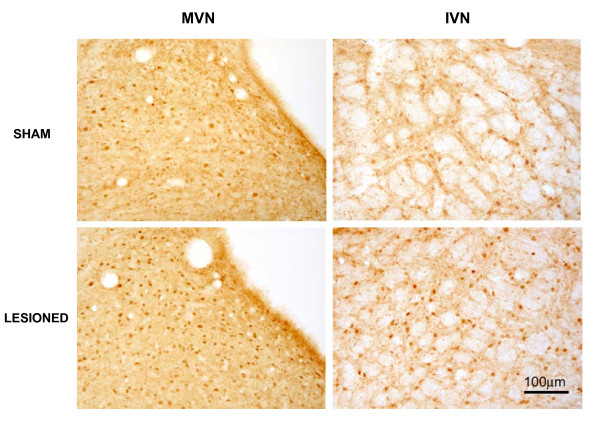
**Illustration of the NF**_**K**_**B immunostaining in the MVN and IVN in a sham rat and in a rat subjected to chemical UL, killed one day after the lesion**. The number of NFκB-Ir cells increased in the MVN and IVN of the UL rat. Scare bar: 100 μm.

Figure 2 shows microphotographs of NFκB-Ir sections taken for the ipsilateral MVN and IVN from one representative sham-operated rat and from one rat killed 1 day after the lesion. As observed with TNFα, the number of NFκB-Ir cells in both VN increased substantially after arsanilate UL.

**Figure 3:**
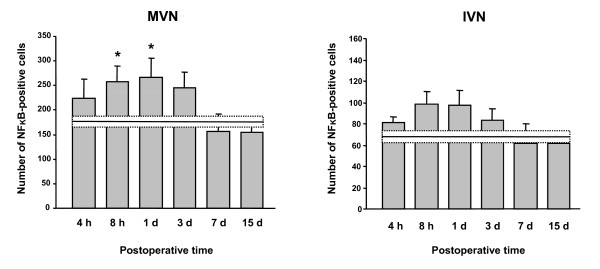
**Quantitative analysis of the effects of chemical UL on NF**_**K**_**B-Ir cells in the MVN and IVN**. Data are mean values (± SEM) of the number of NFκB-Ir cells in the MVN and IVN for rats that had chemical UL and observed at 4, 8 h, 1, 3, 7, and 15 days after the lesion. Data of all postoperative times were pooled in sham animals, and mean value is shown as a horizontal line and standard error of the mean (± SEM) as two dotted lines. According to Bonferroni's principle, P < 0.01 was considered significant. * P < 0.01 versus sham values.

Quantitative analysis of the data is shown in Figure 3 for the MVN and the IVN, displayed here as representative nuclei of the VN complex. The average number of NFκB-Ir cells in the MVN and IVN was 177.1 ± 11.5 and 68.5 ± 5.9, respectively, in the controls. A significant bilateral increase was first observed 8 h postlesion in the MVN (258.2 ± 30.6, p < 0.01) and remained unchanged at 1 day (267.3 ± 37.9 for MVN, p < 0.01). Three days after arsanilate lesion, the mean number of NFκB-Ir cells tend to return to control values in the MVN (244.6 ± 32.0). The same time-course was observed in the IVN, even tough it remained just below the level of significance (p = 0.03 and p = 0.02 at 8 h and 1 day respectively).

#### Chemical UL-induced change in MnSOD immunoreactivity in the vestibular nuclei

Repeated-ANOVA revealed significant effects of the lesion (p < 0.001), the structure (p < 0.0001), and the postoperative time (p < 0.01); it also showed that the interaction between these factors (p < 0.05) was significant. As for NFκB quantification, data obtained in the sham-operated group at the different postoperative times were pooled and expressed as control values.

**Figure 4:**
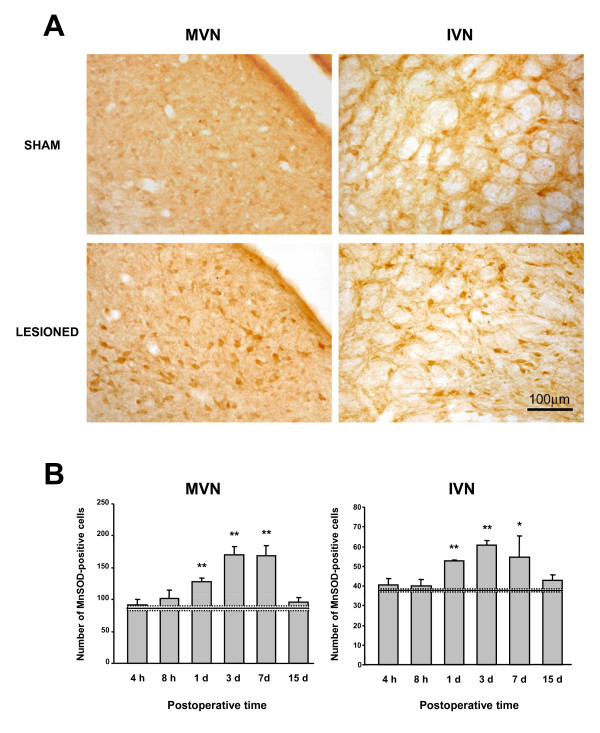
**Chemical UL-induced changes of MnSOD-Ir cells in the MVN and IVN**. (A) Illustration of a typical labeling in a representative sham-operated rat and in a chemical UL animal examined 3 days after the lesion, showing an increased number of MnSOD-Ir cells in both VN. Scare bar: 100 μm. (B) Quantitative analysis of the effects of chemical UL on MnSOD-Ir cells in the MVN and IVN. Data are mean values (± SEM) of the number of MnSOD-Ir cells in the MVN and IVN for rats that had chemical UL and observed at 4, 8 h, 1, 3, 7, and 15 days after the lesion. Data of all the postoperative times were pooled in sham animals, and mean value is shown as a horizontal line and standard error of the mean (± SEM) as two dotted lines. According to Bonferroni's principle, P < 0.0083 was considered significant. * P < 0.0083, ** P < 0.00083 versus sham values.

Arsanilate UL resulted in an increase in MnSOD expression in the bilateral VN. An illustration of this effect in the MVN and IVN, 3 days postoperatively is shown in Figure 4A. This change of MnSOD expression was also observed in the SVN but not in the LVN.

Figure 4B illustrates the quantitative analysis of these data in the MVN and the IVN. Compared to controls, which exhibited a mean number of MnSOD-Ir cells of 86.6 ± 3.7 for the MVN and 37.9 ± 0.8 for IVN, the 1 day postlesion group displayed an increase in MnSOD-labeled cells with a mean number of 127.6 ± 6.1 and 52.8 ± 0.6 for the MVN and IVN respectively (p < 0.00083). The number of MnSOD-Ir cells continued to increase at 3 days postlesion (170.2 ± 13.4 and 60.6 ± 2.4 for the MVN (p < 0.00083) and for the IVN (p < 0.00083) respectively), and remained unchanged in the MVN at 7 days (167.9 ± 17.3, p < 0.00083). Fifteen days after arsanilate UL, normal values were recorded in the MVN (96.4 ± 7.0) and the IVN (42.6 ± 2.9).

#### Upregulation of TNFα, NFκB, and MnSOD expression after chemical and mechanical UL

Repeated ANOVA of the cell counts of TNFα, NFκB, or MnSOD-positive cells revealed no significant effect of the type of lesion (chemical vs mechanical) and no significant interaction between the lesion and the postoperative time for all the markers. No significant differences were found between the ipsilateral and contralateral MVN and IVN of mechanical UL rats at the different postoperative times. Consequently, data from both sides were pooled and averaged for each group of mechanical UL rats.

**Figure 5:**
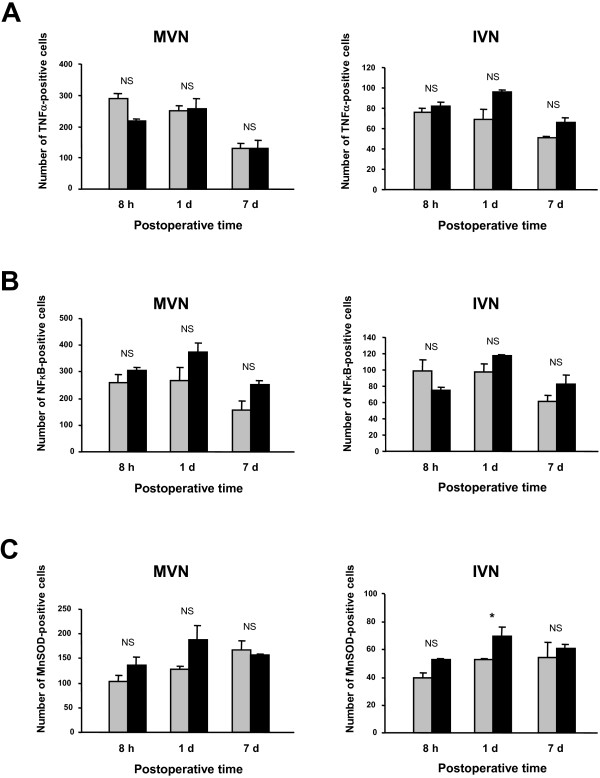
**Comparative TNFα, NF**_**K**_**B and MnSOD immunostaining in the MVN and the IVN after a chemical and a mechanical UL**. Quantitative analysis of the effects of chemical and mechanical UL on TNFα-Ir cells (A), NFκB-Ir cells (B) and MnSOD-Ir (C) in the MVN and IVN. Data are mean values (± SEM) of the number of positive cells recorded in the chemical UL rats (grey histograms) and in the mechanical UL rats (black histograms) observed at 8 h, 1 and 7 days after the lesions. The data from both sides were pooled for all groups of animals. According to Bonferroni's principle, P < 0.016 was considered significant. * P < 0.016 mechanical versus chemical values.

Quantitative analysis of TNFα, NFκB, or MnSOD immunolabeling in the MVN and IVN after mechanical and chemical UL is shown in Figure 5. Whatever the postoperative times, the mean values in the mechanical UL rats did not differ significantly from those of the chemical UL rats (Figure 5).

For the TNFα-positive cells, the mean numbers recorded in the 8 h postoperative group were 288.6 ± 11.9 and 218.5 ± 6.7 in the MVN for the chemical and mechanical UL rats, respectively (NS). A similar upregulation of TNFα-Ir cells was observed with both types of lesions at the 1 day survival period in the MVN (Figure 5A). Similar changes were found in the IVN when considering the chemical and mechanical groups of rats (Figure 5A), and when considering the NFκB-positive cells (Figure 5B) as well as the MnSOD-positive cells (Figure 5C) in both the MVN and IVN. Only one significant difference (p < 0.016) was seen for the MnSOD-positive cells in the IVN at the 1 day survival period, in favor of the mechanical UL group. In addition, return towards normal values was similar for the three markers considered in the present study in both the chemical and mechanical UL rats, indicating a similar time-course of upregulation in both models of unilateral vestibular deafferentation.

### 2. Behavioral investigations

#### Behavioral observations

Unilateral inner ear injection of arsanilate induced the typical behavioral signs of vestibular deafferentation. All arsanilate-UL animals exhibited characteristic oculomotor nystagmus with slow phase directed toward the lesioned side and fast phase beating toward the contralateral side. The postural and locomotor symptoms consisted of rolling and circling behaviors, head tilt and falls toward the lesioned side, and postural asymmetry with strong enhancement of the support surface.

#### Recovery of static posture function

**Figure 6:**
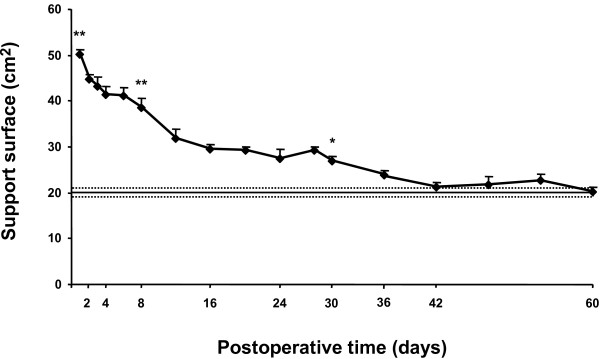
**Recovery of static posture function after chemical UL**. The support surface (expressed in cm^2^) was evaluated for each rat (n = 4 measurements per rat) at regular time intervals until complete recovery. Each point represents the mean value (± SEM) for each postoperative time calculated in a group of chemical UL rats (n = 6). The mean of support surface preoperative values is shown as a horizontal line and standard error of the mean (± SEM) as two dotted lines. Note the strong increase in surface in the days following chemical UL and the complete recovery after 36 days. According to Bonferroni's principle, P < 0.0038 was considered significant. * P < 0.0038, ** P < 0.00038 versus preoperative values.

Recovery of static posture function after arsanilate-UL was evaluated by measuring the support surface delimited by the four legs of the rat standing erect at rest. Figure 6 shows the time-course of posture function recovery after vestibular lesion. The support surface was rather small in normal rats (20.2 ± 1.0 cm^2^) and greatly increased in the days following the vestibular lesion. Maximal support surface increase (50.1 ± 1.1, p < 0.00038) was observed 1 day postlesion, resulting from a strong hypotonia of the limb extensors ipsilateral to the lesion and strong hypertonia of the contralateral extensors muscles. The support surface decreased progressively during the following days but remained superior to preoperative values on the 8^th ^postoperative day (38.6 ± 1.5, p < 0.00038). At 30 days postlesion, the support surface remained significantly larger (27.0 ± 1.2, p < 0.0038). The complete compensation of this postural parameter required 36 days.

#### Recovery of dynamic posture function

Before vestibular lesion, all animals had the ability to right themselves when they were held in a supine position and dropped in the air. The animals also succeeded in flexing their neck and extending their forelimbs when they were lowered toward the ground. The air-righting and the landing reflexes were normal in these controls and the global score was zero.

**Figure 7:**
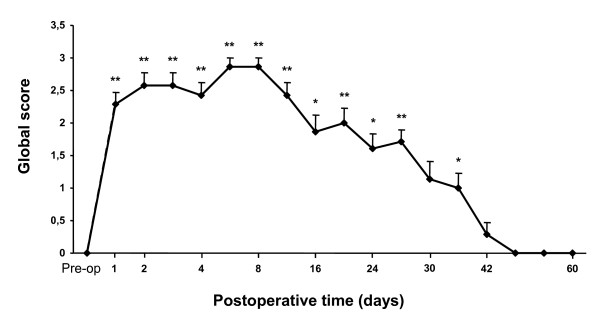
**Recovery of dynamic posture function after chemical UL**. Each point represents the global score of the rats to the air-righting and landing reflex tests at each postoperative time and was compared to preoperative values. A zero score indicated a normal vestibular function, scores ranging from 1 to 4 pointed to vestibular dysfunction and less complete vestibular compensation. Before chemical UL (pre-op), the mean global score was zero. After the lesion, rats failed in performing correct body air-righting and landing reflexes. Recovery was complete 42 days after UL. According to Bonferroni's principle, P < 0.0038 was considered significant. * P < 0.0038, ** P < 0.00038, versus preoperative values.

After arsanilate-UL, rats failed to succeed in either test. Figure 7 shows the mean time-course of recovery. One day after the lesion, the mean global score of the UL rats was 2.3 ± 0.2 (p < 0.00038), indicating that animals failed in performing correct body air-righting reflex and landing reflex. From day 2 to 8, the mean global score remained significantly increased (2.9 ± 0.1, p < 0.00038) on day 8. Then, the UL rats' deficits gradually decreased over time and animals fully recovered both air-righting reflex and landing reflex as correctly as normal rats 42 days after UL only.

## Discussion

The present study shows first that a chemical UL induces the typical postural and locomotor symptoms seen after all types of vestibular deafferentation and a recovery of both static and dynamic posture functions requiring 5-6 weeks. Second, the present findings demonstrate that chemical UL induces a rapid inflammatory reaction in the VN, as shown by the significantly increased expression of the proinflammatory cytokine TNFα. This TNFα upregulation is followed by an increased expression of the antioxidant enzyme MnSOD, associated with the upregulation of the nuclear transcription factor NFκB. Similar changes of TNFα, NFκB, and MnSOD expression were found in rats submitted to mechanical UL. The changes in the expression of neuroprotective factors could constitute a favorable neuronal environment for the vestibular compensation process.

### 1. Behavioral recovery after chemical UL

Unilateral transtympanic injection of sodium arsanilate in the rat induces classical postural deficits (ipsilateral head tilt, increased support surface with ipsilateral limb flexion and contralateral limb extension) and locomotor (ipsilateral falls, rolling and circling) deficits. However, the complete recovery of these postural functions is much longer than that reported in this species after mechanical UL [9,33]. Indeed, head tilt and postural asymmetry disappeared 3-4 days after mechanical UL [9], while 6 weeks were required in the present study. These differences in the time constant of the recovery process were already reported by Kim et al. [34], who showed head deviation peaking 2 h and 3 days after mechanical UL and chemical UL, respectively. In addition, these authors reported a head posture significantly tilted during at least one week after chemical UL since longer recovery delays were not tested in their study. Interestingly, the unilateral section of the vestibular nerve (UVN) in the cat model also induced a delayed behavioral recovery (6 weeks) compared to mechanical UL (2 weeks) [1]. Such differences in the recovery profiles can be accounted for by the nature of the VN deafferentation. There is a total, sudden, anatomical and functional deafferentation after UVN whereas mechanical UL leads to a partial and progressive anatomical deafferentation [1,35]. And it was recently reported that the plasticity mechanisms involved in the recovery process also differ with the type of vestibular deafferentation [36].

A first explanatory hypothesis is that chemical UL is a model of VN deafferentation that is closer to the UVN model. Indeed, intratympanic injection of sodium arsanilate has been shown to cause permanent loss of vestibular function as a result both of degeneration of the vestibular sensory epithelium [37,38] and of the vestibular nerve [39]. This degeneration of the vestibular nerve would explain why the recovery process is longer after chemical UL than after mechanical UL. However, recent findings in the adult rat submitted to chemical UL, similar to our protocol, clearly showed that the Scarpa's ganglion neurons, the vestibular nerve, and the brainstem structures did not exhibit any degeneration after intratympanic injection of sodium arsanilate, from 3 days to 1 month after injection (Chabbert, personal communication). The transtympanic injection targets on epithelial vestibular sense organ (and on cochlear epithelium) and toxicity of arsanilate were similar to that of glutamate excito-toxicity [40]. In this case, the differences between mechanical and chemical UL could be in the postlesion discharge in the vestibular nerve. No electrophysiological inputs to the vestibular nuclei are seen just after mechanical UL while abnormal electrophysiological action potentials could occur in the vestibular nerve after arsanilate lesion, due to axonal terminations still connected to hair cells. This hypothesis is based on the observations of heterotopic electrical discharges in the hair cells after glutamate excito-toxicity [40]. If confirmed, this hypothesis could explain the longer recovery time-course after chemical UL than mechanical UL in rodents.

### 2. Inflammatory and neuroprotective responses in the VN after unilateral vestibular deafferentation

A massive microglial and astroglial reaction was observed in the bilateral VN after mechanical UL in the rat [8,9]. Moreover, axotomy or removal of the peripheral sensory hair cells failed to cause any neuronal cell death in the VN [8]. Not only the second-order vestibular neurons do not degenerate, but these neuronal cells recover a near normal resting discharge one week after mechanical UL in the guinea-pig [41]. The reactive gliosis observed in the VN could be therefore a beneficial mechanism protecting the injured nervous tissue. As suggested by Campos-Torres et al. [9] and described in other nerve injury paradigms [42,43], pro- and anti-inflammatory cytokines produced by activated glial cells could promote the survival of deafferented vestibular neurons and therefore may contribute to the vestibular compensation process.

#### Chemical UL-induced upregulation of TNFα in the VN

Our findings clearly showed a significant increase in the proinflammatory cytokine TNFα in the bilateral VN, detectable as early as 4 h after UL and persisting until 3 days. TNFα is expressed by neurons, in addition to glial cells, in the normal brain, and TNFα receptors are present on all neural cell types, rendering the cells responsive to TNFα signaling [11]. TNFα is upregulated in several neurodegenerative disorders (cerebral malaria, AIDS dementia, Alzheimer's disease) and has been primarily considered to promote the pathologies [44-46]. Besides this potential neurotoxic effect, numerous studies have revealed an unexpected role of TNFα in CNS repair. It protects the brain against ischemia or nitric oxide-induced injury [18,20], and glutamate [17,19,47] or β amyloid neurotoxicity [15,23], in part by induction of antioxidant pathway [15]. Neuronal damage after focal cerebral ischemia or administration of kainic acid is increased in mice lacking TNFα receptor [48]. After optic nerve axotomy, TNFα was found to prevent secondary death of the retinal ganglion cells [16]. Such a neuroprotective effect of TNFα could promote the survival of both the present VN neurons (no cell death) and the newly generated neurons found in the deafferented VN after UVN in the cat [5,6]. The wide variety of responses to TNFα, including apoptosis as well as cell survival and cell proliferation, can be in part explained by signaling pathways mediated through different TNF receptors (TNFR). TNFR1 contains an intracellular "death domain" associated with the detrimental effects of TNFα in models of acute neurodegeneration [13,49,50] while TNFR2 would contribute to cell survival, growth, and proliferation [13,17,51-53].

#### Chemical UL-induced upregulation of NFκB in the VN

NFκB is a transcription factor activated after brain ischemia [54] and spinal cord injury [55,56] and in neurodegenerative processes [57]. However, like TNFα, NFκB also has a neuroprotective role. Preactivation of NFκB by ceramide or by low amounts of TNFα protects neurons from glutamate ototoxicity or oxidative insults [58]. NFκB activation mediates the anti-death action of TNFα in hippocampal neurons exposed to hypoxia or β amyloid toxicity [15,24,28]. Such a TNFα-induced NFκB activation could play a similar neuroprotective action after chemical UL. Indeed, our results showed that NFκB was upregulated in the bilateral VN shortly after the upregulation of TNFα. In cortical neurons exposed to glutamate toxicity, the signaling pathways involve TNFR1 and TNFR2 [17]. While activation of the TNFR1 pathway leads to a transient NFκB upregulation (30 min), TNFR2 signaling promotes persistent and long lasting NFκB activation (up to 24 h) [17]. These authors suggested that the duration of NFκB activation was critical to achieve significant tissue protection. Since NFκB was upregulated in the VN up to 20 h after onset of TNFα upregulation, we hypothesize that TNFα could act through TNFR2 to activate neuroprotective signaling pathways involved in the recovery process after chemical UL.

#### Chemical UL-induced upregulation of MnSOD in the VN

A delayed and long lasting upregulation of MnSOD was shown also in the bilateral VN after chemical UL. These changes were detectable 1 day after arsanilate injection and persisted up to 7 days. MnSOD is regulated transcriptionally and induced by inflammatory cytokines, including TNFα, IL1, and IFNγ [59-61]. Yune et al. [22] demonstrated that exogenous TNFα induces MnSOD expression in the uninjured spinal cord, a process that is inhibited after treatment with a TNFα antibody in injured tissue.

Inflammatory cytokines like TNFα generate reactive oxygen species (ROS), and the mitochondrial respiratory chain is the main source of TNFα-induced ROS [62]. The cells contain a variety of antioxidant enzymes, such as mitochondrial MnSOD, which defend against free radicals damage. MnSOD protects cells against oxidative stress by rapidly dismutating superoxide radicals (O_2_) to hydrogen peroxide (H_2_O_2_) [63]. Hydrogen peroxide can be further detoxified into water and oxygen by catalase or glutathione peroxidase [64]. Accumulating data indicate that MnSOD is critical for neuronal survival against various stress-induced brain injuries [25,65], and that increased levels of TNFα in response to cytotoxic insults protect neurons in part via the induction of cytoprotective genes, including MnSOD [22-24,26]. MnSOD protection has also been demonstrated in non-neuronal cells [21,27,66].

Little is known, however, on the molecular mechanisms that govern transcription of the MnSOD gene. NFκB has been proposed as one of the three transcription factors (with AP-1 and C/EBP-β) regulating TNFα-induced MnSOD expression in different cell types [21-23,62,66]. It is likely that MnSOD induction, found in the present study in VN cells after chemical UL, is mediated through activation of NFκB. However, NFκB and MnSOD expressions showed different kinetics, with NFκB peaking at 1 day postlesion while MnSOD expression peaked 3 days postlesion, as previously reported after spinal cord injury [22]. This suggests that other transcription factors could be involved. Before being activated, NFκB resides in the cytoplasm in an inactive form consisting of three subunits: p50 and p65 (constituting the factor dimer p50-p65) and the IκB inhibitory subunit. When IκB is bound to p50-p65, NFκB is inactive. Signals activating NFκB (TNFα: see [67]) cause dissociation of this complex, releasing p50-p65 that then translocates to the nucleus and binds to specific κB DNA sequences in the promoter region of a variety of NFκB-responsive genes, such as MnSOD gene. Xu et al. [27] found that induction of MnSOD by TNFα required the presence of an intronic nuclear factor κB (NFκB) site in the MnSOD gene. And protection of neuronal cells from β-amyloid peptide toxicity by TNFα-induced MnSOD activation resulted from an enhanced binding of the dimer p50-p65 to the promoter/enhancer regions of the MnSOD gene [23]. The upregulation of the neuroprotective MnSOD protein observed in our chemical UL model could be due to activation of such a sequence.

#### Effects of chemical and mechanical UL on TNFα, NFκB, and MnSOD upregulation in the rat

Comparison of chemical and mechanical UL data strongly suggests that the inflammatory response observed after chemical UL is not due to a toxic effect per se of sodium arsanilate on brainstem structures. Since the amount as well the kinetics of the upregulation of the three markers did not differ for the mechanical UL group compared to the chemical UL group, this suggests that the cellular response to vestibular deafferentation is similar in both models.

These results are supported by Campos-Torres and de Kazak's personal observations, reported in the discussion of the paper by Campos-Torres et al., 2005 [9]. The authors studied cytokine expression within the vestibular nuclei at different times after mechanical labyrinthectomy in the rat. They reported that the real time light cycler RT-PCR studies showed a significant upregulation of TNFα and interleukin (IL1, IL-6) within the vestibular nuclei beginning at 6 h after the lesion.

#### Changes of TNFα, NFκB and MnSOD protein in the bilateral VN

Why the changes in all the markers (TNFα, NFκB, and MnSOD) can be seen bilaterally, given that the lesion was unilateral, can be explained with respect to the neural connectivity between the VN on both sides, and to their cell content. Neuroanatomical studies have shown that the VN neurons are under the control of both excitatory inputs from the ipsilateral labyrinth and inhibitory inputs from the contralateral labyrinth via direct projections (commissural system) and indirect ones (cerebellar cortex loops) [68]. It was also shown that the VN contains different subpopulations of neurons, including excitatory second order vestibular cells (tonic and kinetic type I neurons), inhibitory second order vestibular cells, excitatory interneurons, and inhibitory interneurons (type II neurons), at least for the MVN [69]. Vestibular deafferentation can therefore lead to changes in different subpopulations of cells in the VN on the intact and lesioned sides. Dysinhibitory and/or dysfacilitatory processes can activate type I cells on the intact side, as evidenced electrophysiologically [70], whereas facilitatory processes can activate both excitatory and inhibitory interneurons on the lesioned side. Such mechanisms can also explain the early bilateral increase in c-Fos mRNA and Fos protein levels observed in the MVN, SVN, and IVN after hemilabyrinthectomy in the rat [71]. Indeed, those authors observed that the changes were asymmetric in the MVN, being most prominent in the dorsal part of the contralateral MVN (where second order vestibular neurons are located) and in the ventral part of the ipsilateral nucleus (where commissural neurons acting on the MVN of the intact side are located). Many other works also pointed out bilateral changes in the cat model for c-Fos [72], choline acetyltransferase-Ir [73], and GABA-Ir [[74], see 36 for a review].

## Conclusions

Our study indicates that behavioral recovery after chemical UL in the rat is similar to that previously reported in the cat model after UVN. We provide cellular evidence that this type of vestibular deafferentation, as well as mechanical UL, induces a strong inflammatory reaction in the VN, as shown by the increased expression of the proinflammatory cytokine TNFα. The compensatory responses to this stress, that is, upregulation of the neuroprotective factors NFκB and MnSOD, very likely constitute a favorable environment 1) to prevent existing VN cells from apoptosis, and 2) to promote the plasticity mechanisms involved in the recovery process, including cell survival and cell differentiation of newly generated cells. Indeed, the role of TNFα and NFκB in cell proliferation and cell differentiation has been strengthened by recent reports in both neonatal [75] and adult [31,76] brain structures. The present findings strongly suggest that proinflammatory cytokines and induced neuroprotective factors play a role in the recovery of vestibular functions.

## List of abbreviations used

ANOVA: analysis of variance; BSA: bovine serum albumin; IVN: inferior vestibular nucleus; LVN: lateral vestibular nucleus; MVN: medial vestibular nucleus; NFκB: nuclear transcription factor κB; MnSOD: manganese superoxide dismutase; SVN: superior vestibular nucleus; TNFα: tumor necrosis factor alpha; PB: phosphate buffer; PBS: phosphate buffer saline; UL: unilateral labyrinthectomy; UVN: unilateral vestibular neurectomy; VN: vestibular nuclei.

## Competing interests

The authors declare that they have no competing interests.

## Authors' contributions

ML(*) is the main investigator of this work in charge of the study design, analysis and interpretation of results, and writing. CM and LBD both participated in immunohistological investigations and analysis of the data. ML(**) participated mainly in the writing of the report. All authors read and approved the final manuscript.
